# Exploring the Complementarity of Pancreatic Ductal Adenocarcinoma Preclinical Models

**DOI:** 10.3390/cancers13102473

**Published:** 2021-05-19

**Authors:** Owen Hoare, Nicolas Fraunhoffer, Abdessamad Elkaoutari, Odile Gayet, Martin Bigonnet, Julie Roques, Rémy Nicolle, Colin McGuckin, Nico Forraz, Emilie Sohier, Laurie Tonon, Pauline Wajda, Sandrine Boyault, Valéry Attignon, Séverine Tabone-Eglinger, Sandrine Barbier, Caroline Mignard, Olivier Duchamp, Juan Iovanna, Nelson J. Dusetti

**Affiliations:** 1 Cancer Research Center of Marseille, CRCM, Inserm, CNRS, Paoli-Calmettes Institut, Aix-Marseille University, 13288 Marseille, France; owen.hoare@inserm.fr (O.H.); nicolas.fraunhoffer@inserm.fr (N.F.); abdessamad.elkaoutari@inserm.fr (A.E.); odile.gayet@inserm.fr (O.G.); martin.bigonnet@inserm.fr (M.B.); julie.roques@inserm.fr (J.R.); 2 Tumour Identity Card Program (CIT), French League Against Cancer, 75013 Paris, France; remy.nicolle@ligue-cancer.net; 3 CTIBIOTECH, Cell Therapy Research Institute, 69330 Lyon, France; c.mcguckin@ctibiotech.com (C.M.); nico.forraz@ctibiotech.com (N.F.); 4 Centre Léon Bérard, 69008 Lyon, France; Emilie.SOHIER@lyon.unicancer.fr (E.S.); laurie.tonon@lyon.unicancer.fr (L.T.); Pauline.WAJDA@lyon.unicancer.fr (P.W.); sandrine.boyault@lyon.unicancer.fr (S.B.); Valery.Attignon@lyon.unicancer.fr (V.A.); Severine.TABONE-EGLINGER@lyon.unicancer.fr (S.T.-E.); 5 Ipsen Innovation, 91940 Les Ulis, France; sandrine.barbier@ipsen.com; 6 Oncodesign, 21000 Dijon, France; cmignard@oncodesign.com (C.M.); oduchamp@oncodesign.com (O.D.); 7 Paoli-Calmettes Institut, 13009 Marseille, France

**Keywords:** pancreatic cancer, chemosensitivity prediction, personalized medicine, in vivo models, in vitro models

## Abstract

**Simple Summary:**

Pancreatic ductal adenocarcinoma (PDAC) patient care lacks well-established molecular characterization of the tumors, which would allow for better-personalized treatment selection if improved. To overcome this problem, preclinical models are frequent-ly adopted tools used to elucidate the molecular characterization of PDAC tumors. Unfortunately, the vast majority of studies using these preclinical models fail when transferred to patients despite initially promising results. This study presents for the first time a comparison between three preclinical matched models directly derived from patient tumors. We show that their applicability to preclinical studies should be considered with a complementary focus, avoiding tumor-based direct extrapolations, which might generate misleading conclusions and consequently the overlook of clinically relevant features. We finally highlight the importance of validating and refining predictive transcriptomic signatures using a combination of these models.

**Abstract:**

*Purpose*: Compare pancreatic ductal adenocarcinoma (PDAC), preclinical models, by their transcriptome and drug response landscapes to evaluate their complementarity. *Experimental Design*: Three paired PDAC preclinical models—patient-derived xenografts (PDX), xenograft-derived pancreatic organoids (XDPO) and xenograft-derived primary cell cultures (XDPCC)—were derived from 20 patients and analyzed at the transcriptomic and chemosensitivity level. Transcriptomic characterization was performed using the basal-like/classical subtyping and the PDAC molecular gradient (PAMG). Chemosensitivity for gemcitabine, irinotecan, 5-fluorouracil and oxaliplatin was established and the associated biological pathways were determined using independent component analysis (ICA) on the transcriptome of each model. The selection criteria used to identify the different components was the chemosensitivity score (CSS) found for each drug in each model. *Results*: PDX was the most dispersed model whereas XDPO and XDPCC were mainly classical and basal-like, respectively. Chemosensitivity scoring determines that PDX and XDPO display a positive correlation for three out of four drugs tested, whereas PDX and XDPCC did not correlate. No match was observed for each tumor chemosensitivity in the different models. Finally, pathway analysis shows a significant association between PDX and XDPO for the chemosensitivity-associated pathways and PDX and XDPCC for the chemoresistance-associated pathways. *Conclusions*: Each PDAC preclinical model possesses a unique basal-like/classical transcriptomic phenotype that strongly influences their global chemosensitivity. Each preclinical model is imperfect but complementary, suggesting that a more representative approach of the clinical reality could be obtained by combining them. *Translational Relevance*: The identification of molecular signatures that underpin drug sensitivity to chemotherapy in PDAC remains clinically challenging. Importantly, the vast majority of studies using preclinical in vivo and in vitro models fail when transferred to patients in a clinical setting despite initially promising results. This study presents for the first time a comparison between three preclinical models directly derived from the same patients. We show that their applicability to preclinical studies should be considered with a complementary focus, avoiding tumor-based direct extrapolations, which might generate misleading conclusions and consequently the overlook of clinically relevant features.

## 1. Introduction

Pancreatic ductal adenocarcinoma (PDAC) is one of the most lethal of all adult malignancies, with an extremely poor survival rate [1]. However, the mortality rate associated with the disease is anticipated to surge in the coming years as the second most common cause of cancer-related deaths [2]. Approximately, 18% of patients achieve a 1-year survival rate [3] and only 7% achieve a 5-year survival rate across all stages [4]. Clinical manifestations of PDAC often present with metastatic disease and consequently patient outlook is extremely poor [5]. Moreover, 15–20% of pancreatic cancer patients are deemed resectable whilst approximately 30% are borderline resectable [6]. Thus chemotherapy is the only available option to improve clinical outcomes for patients who have an unresectable disease [7].

Transcriptomic unsupervised analysis of PDAC identified multiple subgroups of the disease associated with unique biological features and prognostic outcomes [8]. Two different subtypes have been mainly described: the classical and the basal-like [9,10,11]. Briefly, the classical tumors are characterized as having a high immune infiltrate, better response to chemotherapy and improved survival; the basal-like have a worse prognosis and are more resistant to chemotherapy [12]. These subtypes were also identified in patient-derived xenografts (PDX) with specific markers for the basal-like and classical subtypes [13]. Recently a binary classifier, purity independent subtyping of tumors (PurIST), was described, harmonizing the previous classifications [14]. However, these discrete stratifications were challenged by the presence of multiple subtypes within the same tumor, demonstrating a clinical need to grade the PDAC tumors as a continuous gradient [15,16]. Given that these different phenotypes greatly influence response to treatment it is important to consider these in the context of chemosensitivity studies.

Accurately quantifying drug sensitivity that mimics the patient’s response to routinely used chemotherapeutics in preclinical models is essential to ameliorate clinical outcomes. This would also help in identifying novel molecules to target the chemo-resistant phenotypes improving in this way the abysmal prognosis associated with the disease [17]. Currently, the underlining mechanisms of drug resistance remain elusive and a challenging obstacle to overcome clinically [18]. To solve this problem some preclinical models closely derived from patient tumors have been developed. Among them, PDX has been shown to faithfully recapitulate the histology, genetic profile, drug response, and heterogeneity of primary human tumors [19] and is largely considered the best model for predicting clinical response [19,20]. Despite the obvious advantages, PDX is not always the preferred model given the cost and labor involved in maintaining them [21,22]. Incidentally, very frequently employed PDAC patient-derived preclinical models for drug screening are in vitro 2D and 3D cultures such as primary cell cultures and patient derived organoids, respectively. However, the strategy orienting the decision on which type of preclinical model to choose and/or how to combine them in studying the patient tumor chemosensitivity response remains unclear. Are these models redundant or complementary? Do we need to choose the best of them or a combination of them in order to circumvent their individual weaknesses? A French consortium, named Innovative MODels Initiatives (IMODI), was organized aiming to analyze the advantages and limitations of the main cancer preclinical models. The aim of our work was to produce paired PDX, primary cell cultures and patient derived organoids to compare their transcriptome and drug response landscapes evaluating through them their complementarity. We conclude that each of these preclinical models is imperfect but complementary. A more representative response of the reality could be obtained by combining them.

## 2. Materials and Methods

### 2.1. RNA-Sequencing Data Analysis

Twenty PDX samples were obtained and 19 underwent RNA-sequencing. In the case of xenograft-derived pancreatic organoids (XDPO), 17 grew well in culture and underwent RNA-sequencing and for the xenograft-derived primary cell cultures (XDPCC), 17 grew well in culture and 16 underwent RNA-sequencing (Table S1). RNA was extracted from chemotherapy naïve samples. PDX RNA extraction was done as previously described by Duconseil et al. [23], XDPO as previously described by Fraunhoffer et al. [24] and XDPCC as previously described by Nicolle et al. [13]. Briefly: total RNA was extracted using RNeasy mini kit (Qiagen, Courtaboeuf, France), mRNA profiles were obtained using the TrueSeq Stranded mRNA LT protocol (Illumina, Evry, France). Sequencing followed oligo-dT capture and was done on a paired-end flow cell. mRNA libraries were prepared and sequenced by AROS Applied Biotechnology A/S (Aarhus, Denmark). RNAseq reads were mapped using STAR 18 with the proposed ENCODE parameters and SMAP on the human hg19 and mouse mmu38 genomes and transcript annotation (Ensembl 75). Gene expression profiles were obtained using FeatureCount. PDX, XDPO and XDPCC transcriptomics data were treated separately and normalized independently for all analysis in this study unless otherwise specified. Gene counts were normalized using the 75% upper quantile normalization and log_2_ transformed. The PAMG and PurIST classifier were applied to determine the basal-like and classical phenotypes across the three transcriptomic dataset by merging and normalizing the models together. Furthermore, model proximity was estimated with the top basal and classical markers (+/− 2-fold change and FDR < 0.05) [13], applying principal component assay (PCA) and hierarchical clustering.

### 2.2. Establishment of PDX and In Vivo Chemosensitivity Profiling

The 20 PDX used in this investigation form part of the project IMODI (Innovative MODels Initiative) and were established as previously described by Nicolle et al. [13]. Patients were from the PaCaOmics clinical trial (Clinical Trials.gov: NCT01692873). In vivo replicates for each condition varied between 6 and 10 mice, depending on the successful growth. PDX was allowed to grow until a volume of 200 mm^3^ was reached at which point the mice received the treatment into the tail vein. Control mice received a solution of NaCl 0,9%, irinotecan was given every second day for a total of three 22 mg/kg administrations (Q2Dx3), gemcitabine every third day with a total of four 120 mg/kg administrations (Q3dX4), 5-fluorouracil (5-FU) every four days with a total of two 56 mg/kg administrations (Q4dX2) and oxaliplatin every four days with a total of two 5 mg/kg administrations (Q4dX2). Tumor volumes were measured over a number of days ranging from 0–200 days with a Vernier caliper device twice weekly. The tumor volume was calculated using the following formula, v = (length/width^2^)/2. The doubling time was considered as the time in days that take a tumor to double its volume. Any mice exceeding a tumor volume greater than 2000 mm^3^ were sacrificed and excluded from the experiment for ethical reasons. Several models with multiple treatments do not produce chemograms, they were removed from the study and indicated as “NA” in the Figures and Tables. The drug response data was plotted with the tumor volume increase over the number of days in the experiment using the Prism software. All replicates in the control and treated groups were plotted using non-linear regression and fitted to a sigmoid curve by extrapolating the data to make the best fit curve. Furthermore, to quantitatively compare responses to different treatments, we calculated the area under the curve (AUC) values for responses to each drug taken at the same number of days for both the control and treatment. Each PDX was scored independently, where the treatment AUC was divided by the control AUC and expressed as a percentage to determine the level of chemosensitivity. These were subsequently used as chemosensitivity scores (CSS) for each PDX. This chemosensitivity profiles scoring criteria was chosen because it allows a good comparison between in vivo and in vitro models (see below).

### 2.3. Establishment of XDPCC and XDPO and In Vitro Chemosensitivity Profiling

XDPCC models were originally derived from the same 20 PDX patient models as previously described by Duconseil et al. [23]. To obtain organoids from PDTX, xenografts were split into several small pieces and processed in a biosafety chamber and after a fine mincing, they were treated with the Tumor Dissociation Kit (Miltenyi Biotec, Miltenyi Biotec, Paris, France). Undigested pellets were digested with accutase (Sigma-Aldrich Lyon, France) at 37°C for 30 min. The pancreatic tissue slurry was transferred into a tissue strainer 100 μm and was placed into 12-well plate coated with 150 μL GFR matrigel (Corning, Boulogne-Billancourt, France). The samples cultured with Pancreatic Organoid Feeding Media (POFM) consisted of Advanced DMEM/F12 supplemented with 10 mM HEPES (Thermo Fisher Scientifics, Courtaboeuf, France); 1× Glutamax (Thermo-Fisher Scientifics); penicillin/streptomycin (Thermo-Fisher Scientifics); 100 ng/mL Animal-Free Recombinant Human FGF10 (Peprotech, Peprotech, Neuilly-Sur-Seine, France); 50 ng/mL Animal-Free Recombinant Human EGF (Peprotech); 100 ng/mL Recombinant Human Noggin (Biotechne, Bio-Techne, Rennes, France); Wnt3a-conditioned medium (30% *v*/*v*); RSPO1-conditioned medium (10% *v*/*v*); 10 nM human Gastrin 1 (Sigma-Aldrich Lyon, France) 10 mM Nicotinamide (Sigma Aldrich); 1.25 mM N acetylcysteine (Sigma Aldrich); 1× B27 (Invitrogen, (Invitrogen, Villebon sur Yvette, France); 500 nM A83-01 (Tocris, Noyal Châtillon sur Seiche, France); 10.5 μM Y27632 (Tocris). The plates were incubated at 37 °C in a 5% CO_2_ incubator, and the media were changed every 3 or 4 days. For chemosensitivity profiling, XDPO and XDPCC were plated into 96 well plates and then subjected to incrementally increasing concentrations of drugs (from 1 nM to 1 mM for gemcitabine, 5-FU and oxaliplatin and from 0.1 nM to 0.1 mM for SN38, the irinotecan metabolite). XDPO and XDPCC experiments were performed separately. XDPCC cell viability was measured 72 h after treatment using Prestoblue (Thermo Fisher, Waltham, MA, USA) and Cell Titer Glow 3D (Promega Corporation, Madison, WI, USA) was used for XDPO. Four doubling time calculation (DT) XDPO and XDPCC viability for untreated control conditions was measured on days 0 and 3. The ratio of day 3 over day 0 values corresponds to the replication rate (RR) of the cells in 72 h. Doubling time was calculated with the formula 72*2/RR. Fluorescence and luminescence values were quantified using the plate reader Tristar LB941 (Berthold Technologies, Bad Wildbad, Germany). Each experiment was performed at least 3 times with at least 3 replicates. The drug response (or growth rate inhibition) for XDPO and XDPCC were fitted to a sigmoid curve over the range of doses and sensitivity scores were calculated using the GRmetrics R package [25]. For this study, the AUC of XDPO and XDPCC were used as a chemosensitivity score (CSS) to compare drug resistance across all models due to the closer compatibility with the PDX’s scoring methods.

### 2.4. Identification of Chemosensitivity Transcriptomic Profiles

The top 10,000 most variable genes were selected for the untreated PDX, XDPO and XDPCC datasets separately using the inter-quartile range to perform independent component analysis (ICA) using the JADE algorithm. A total of 13 components were constructed for PDX and XDPCC and a total of 16 components for XDPO. For each drug and model the biologically relevant component that best explained the chemosensitivity was identified by applying a Pearson correlation using the AUC with the top 10,000 genes. The component with the highest correlation and lowest *p*-value was deemed to be the best component. Only genes that have an absolute contribution of 2.5 were selected to generate the heatmaps.

### 2.5. Pathway and Gene Set Enrichment Analysis

Pathway enrichment analysis was performed using the Molecular Signatures Database (MSigDB) on a pre-ranked list of genes from the best component to explain each model’s CSS profile. The 10,000 genes with projection values were used to make the enrichment and only pathways with a *p* < 0.05 were retained. The normalized enrichment score (NES) was used to determine sensitive/resistant pathways. Pathways with positive NES where the component was positively correlated with the AUC are associated with resistance. Conversely, pathways with positive NES where the component was anti-correlated with the AUC are associated with sensitivity and vice versa. Venn Diagrams were constructed comparing the global pathway overlap for all three models and the specific pathways for the sensitive and resistant profiles.

## 3. Results

### 3.1. Transcriptomic Comparison between In Vitro and In Vivo Patient-Derived PDAC Preclinical Models

**Figure 1:**
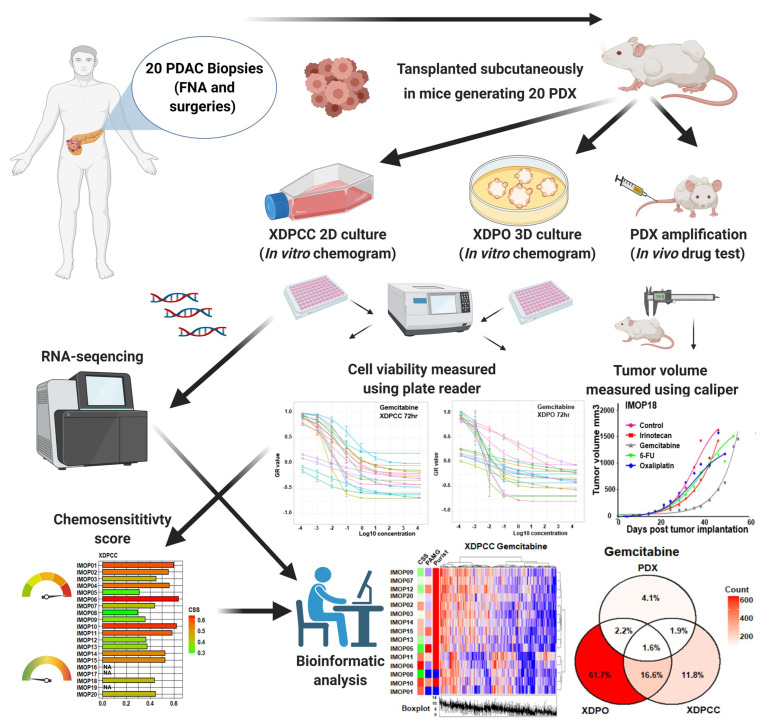
Study workflow. 20 PDAC tumors including surgical resections and fine needle aspirated (FNE) biopsy were subcutaneously transplanted in mice and allowed to grow. From these 20 PDX samples, XDPO and XDPCC were also derived and amplified in culture. Mice were later treated with chemotherapeutics, which were administered intravenously into the tail vein of the mouse. Measurements were taken periodically at set time intervals using a caliper device. Chemosensitivity testing was performed on PDX, XDPO and XDPCC samples and each model also underwent RNA-sequencing for further transcriptional downstream analysis.

**Figure 2:**
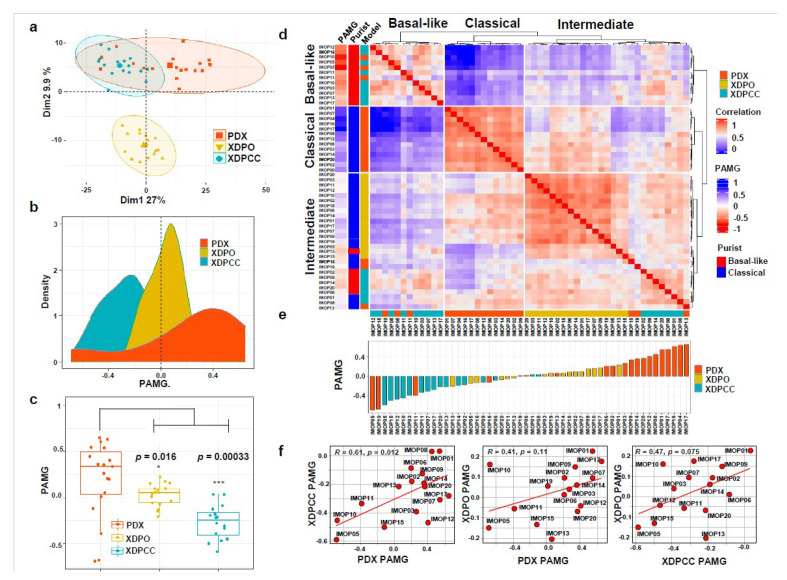
Phenotypic analysis of PDX, XDPO and XDPCC. (**a**) A PCA plot was generated with the top 634 basal-like and classical markers across all three models. (**b**) Density plot showing the distribution of the PAMG across all three models. (**c**) Boxplot and Wilcoxon t-test illustrating the PAMG profile of each model examined. (**d**) ComplexHeatmap with correlation matrix using the 634 top basal-like and classical markers. Annotations to the left and right of the heatmap indicate, the Molecular Grade (PAMG), the PurIST classification and the model type. (**e**) Ranking of the Molecular Gradient (PAMG) across all three models from highest to lowest. Higher values indicate more classical and lower values indicate more basal-like. (**f**) Correlation co-efficient plots generated comparing all models using the PAMG, regression values and *p*-values.

To study the phenotypic characteristics of the three types of patient-derived PDAC preclinical models, we derived: 20 PDX, and from them 17 XDPO and 17 XDPCC. Table S2 shows the clinical characteristic of these patients. First, PDAC were grafted in mice as PDX and then, from PDX, were derived XDPO and XDPCC (Figure 1). The transcriptome of 19 PDX, 17 XDPO and 16 XDPCC untreated models were analyzed (Table S1). Model establishment and characterization were done as part of the IMODI project based on the development of animal models and cell-based assays (Figure 1). Initially, we stratified the three models based on the basal-like/classical classification. A PCA plot was generated using the 634 top basal-like and classical transcriptomic markers identified by Nicolle et al. [13]. PDX showed a greater dispersion capturing more variation in the basal-like and classical phenotypes, whereas XDPO and XDPCC displayed more enrichment in one specific phenotype, tending to be either mostly classical or mostly basal-like, respectively (Figure 2a), with the XDPO as the most homogeneous group. To further characterize the transcriptomes of the models, the PAMG signature was used. PDX had the highest variation in the PAMG continuum, followed by XDPCC and XDPO presenting the lowest degree of variation, conserving the previous tendency observed with the PCA (Figure 2b,c). Similar results were obtained using the top 10,000 genes extracted from the integrated data set (Figure S1a,b), validating that the basal-like/classical transcriptional markers strongly influence the PDAC phenotype determination, independently of the model. Interestingly, XDPO displayed an intermediate PAMG, enriched in classical gene expression (Figure 2d,e). Lastly, we analyzed the correspondence level among models following the PAMG signature (Figure 2f). As expected, XDPCC and PDX displayed the highest correlation (R = 0.61 and *p* = 0.012), whereas XDPO did not correlate significantly with any model, indicating a strong influence of the PAMG dispersion range within the analysis. However, we observed an inter-model positive trend, suggesting high stability of the most extreme patients such as IMOP05 and IMOP01.

### 3.2. Chemosensitivity Profile Scoring and Comparison in the Different Type of Models

**Figure 3:**
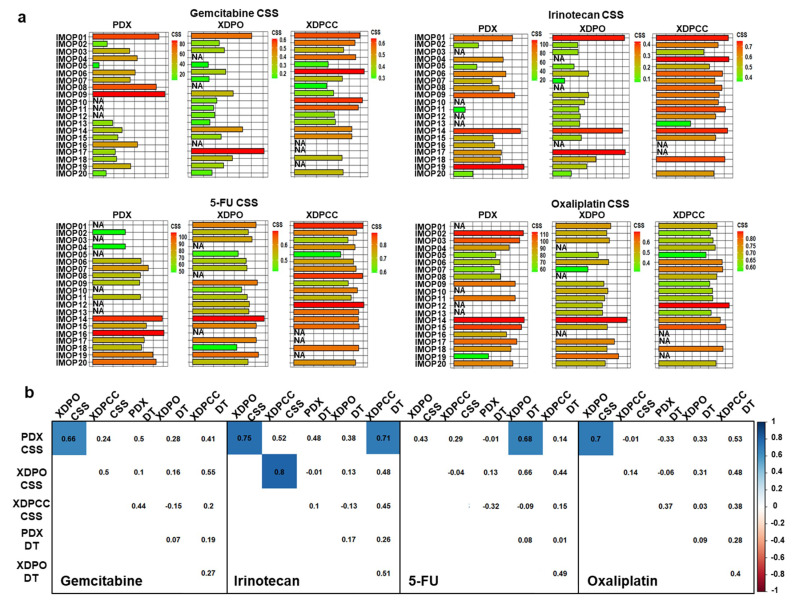
Chemosensitivity score for PDX, XDPO and XDPCC. (**a**) Barplots were generated comparing the chemosensitivity score (CSS) of all models (IMOP01–IMOP20) treated with gemcitabine, irinotecan, 5-FU, and oxaliplatin respectively. Bars in red with a higher CSS indicate more resistance and bars in green with a lower CSS indicate more sensitivity. Non-available (NA) values for some models show no bars. (**b**) Correlation plots comparing the drug sensitivity profile and mean doubling time of all models for gemcitabine, irinotecan, 5-FU, and oxaliplatin. Statistically significant correlations are highlighted in blue squares as *p*-values of 0.05 or less.

PDX chemosensitivity scores were determined as the ratio between the control and the treated group AUC at a fixed dose (Figure S2). Otherwise, XDPO and XDPCC chemograms were performed at multiple doses with 72 h as the endpoint (Figures S3 and S4). Overall, gemcitabine induces the highest response followed by irinotecan, 5-fluorouracil (5-FU), and lastly, oxaliplatin (Figure S5). XDPO were highly variable between experiments and showed a more sensitive profile than the XDPCC (Figures S3 and S5). Interestingly, patient inter-model chemosensitivity heterogeneity was consistent with the model global transcriptomic profile, indicating a strong phenotype dependency (Figure 3a). A correlation of all drug sensitivity scores across all three models was also generated, including the doubling time (DT) to determine if CSS is influenced by the proliferation rate (Figure 3b). For gemcitabine, a significant correlation (R = 0.66, *p* ≤ 0.05) was observed between PDX and XDPO, while PDX and XDPCC did not correlate. Moreover, significant correlations were also observed between PDX and XDPO, for irinotecan (R = 0.76, *p* ≤ 0.05) and oxaliplatin (R = 0.70, *p* ≤ 0.05). These results suggest a relationship between PDX and XDPO chemosensitivity, confirming this models reliability to recapitulate the complex molecular interactions that determine the drug response observed in vivo. Finally, the DT did not correlate with the chemosensitivity for any model, reaffirming the concept that the global phenotype determines the drug response.

### 3.3. Biological Analysis of Chemosensitivity in the Different Type of Models

**Figure 4:**
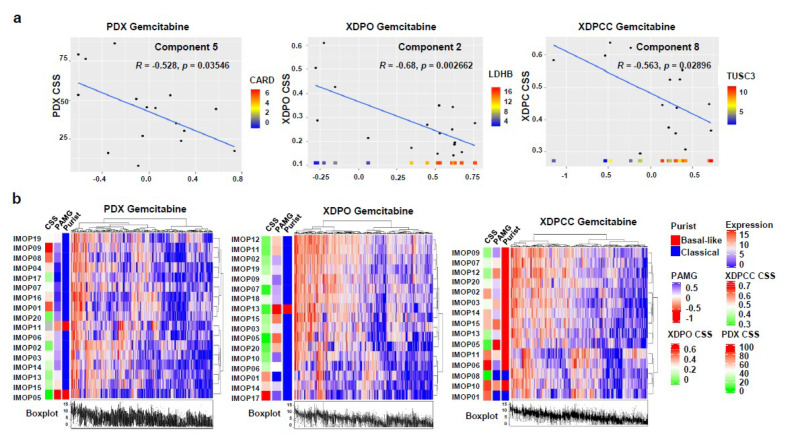
Gemcitabine chemosensitivity profiles using ICA. (**a**) Correlation graph between the best component obtained from the ICA and gemcitabine CSS for PDX, XDPO and XDPCC models. The CSS is displayed on the *y*-axis and the contribution of the witness gene of the *x*-axis. All three models show an anti-correlation. Regression (R) values and p-values are displayed. (**b**) Hierarchical clustering for PDX, XDPO and XDPCC gemcitabine sensitivity components are shown respectively. Rows are clustered using model IDs and columns show the clustering of genes. Boxplots located at the bottom of the heatmap show the variation in expression levels for each gene within the component. Annotations to the right and left of the heatmap indicate, the Molecular Grade (PAMG), the PurIST classification and the model type including CSS. A higher CSS indicates more resistance in red and a lower CSS indicates more sensitivity in green. For the Molecular Gradient (PAMG) a higher value means a more classical phenotype (**blue**) and lower values are a more basal-like phenotype (**red**). Binary classification is also provided using the PurIST method for determining the phenotype where red is basal-like and blue is classical.

**Figure 5:**
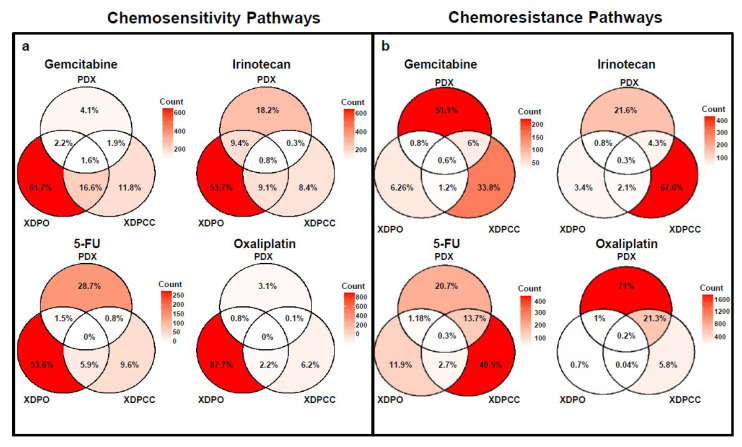
Molecular pathway analysis. Venn diagrams of all molecular pathways in common with all three models. (**a**) chemosensitivity associated pathways, (**b**) chemoresistance associated pathways. Count legend to the right depicts the number of pathways in common between models. The percentages are also included.

To identify transcriptional characteristics that could explain PDX, XDPO and XDPCC chemosensitivity profiles, independent component analysis (ICA) was carried out with the top 10,000 most variable genes for each expression dataset. We determine specific transcriptomic profiles associated with each drug for the three models (Figure 4a, Figures S6a, S7a and S8a). Component 2 for XDPO and component 9 for XDPCC explained more than one drug response, suggesting common intra-model drug response mechanisms independently of the drug type. Component reliability was confirmed through the patient clustering, applying the highest contributing genes, where a clear separation between high and low CSS was observed (Figure 4b, Figures S6b, S7b, and S8b). Finally, pathway enrichment analysis was performed to determine the biological proximity between models linking with the drug response (Figure 5 and Figure S9). Specifically, we discriminate between sensitive and resistant pathways according to the normalized enrichment scores (NES), following each component’s correlation coefficient sign. XDPO pathways for all the drugs were associated with a more sensitive profile and XDPCC pathways displayed the opposite behavior with a more resistant profile enrichment. This tendency impacted their overlap with the PDX, observing a higher intersection with XDPO in the sensitive pathways and XDPCC showing more resistant pathway overlap with PDX (Figure 5 and Supplementary Data File). Overall, these results consolidate our initial results regarding the basal-like/classical phenotype, evidencing a strong correlation between the classical and the basal-like profile with high and low drug response, respectively.

## 4. Discussion

Traditional PDAC preclinical models consist of genetically engineered mice using in situ tumors, established cell lines, transplantation models in which cells are injected into immune-deficient mice and other models directly derived from the patients tumor such as PDX, primary cell cultures and patient-derived organoids. Although all of these models have contributed to a better understanding of PDAC tumor biology and progression, the translation of this knowledge for patient benefit has been weak. In this regard, a potential contributing factor is represented by the inappropriate use of some of these preclinical models. Particular attention has been paid to models derived closely from the patient’s tumor because they predict therapeutic responses better than other models. For this reason, this work was focalized on these models studying their complementarity. We then compared the transcriptome and the chemosensitivity of PDX and their derived organoids and cell lines.

Transcriptome and chemosensitivity are hugely impacted by different factors associated with the establishment of each particular model such as the stroma present within the in vivo models, media used, growth factors, the number of passages, storage conditions and clonal diversification for in vitro models [26]. Despite this fact, we observed a clear transcriptomic phenotypic determination among the models with the PDX representing both classical and basal-like whereas XDPO and XDPCC showed a polarized phenotype toward classical and basal-like, respectively. Consequently, successive evaluations of the transcriptomic phenotype reveal a high concordance between the basal-like PDX and XDPCC and the classical PDX and XDPO. Previous studies have determined a differential drug response associated with the PDAC subtype, positioning the classical tumors as less refractory to the treatment in relation to the basal-like subtype [15]. This observation was also validated in our study, hypothesizing a chemosensitivity profile divergence among the models associated with their subtype capture range. As expected XDPCC with a basal-like profile and a low PAMG was the model less sensitive to chemotherapy, in contrast, PDX and XDPO that are mainly classical displayed a more sensitive profile for all drugs. It was also discovered that there was a statistically significant correlation between the PDX and XDPO models in their drug sensitivity profile in line with previous findings [27,28,29], 5-FU was the only exception and no correlation was reported between PDX and XDPCC chemosensitivity for any drugs. This could be related to the fact that most PDX (70%) and XDPO (more than 90%) are classical.

Importantly, differences can be seen in chemosensitivity for each drug across all models allowing the establishment of a robust CSS. Of note, some patient samples can maintain their sensitivity from one model to the other whilst other samples completely change. However, at the transcriptional level, we observed a strong inter-model consistency to capture the chemosensitivity phenotypes. This transcriptomic stability was confined within the global chemosensitivity model range and was independent in most cases from the PDX source. This fact suggests that each preclinical model can be used for chemosensitivity studies without considering the sensitivity or the resistance obtained in one of the others. This fact reaffirms their use in a complementarity fashion, where one can be used to validate and reinforce the results of the others. Our results do not allow to identify the best model combination, PDX seem to be the most powerful model because it preserves better the transcriptomic inter-tumor heterogeneity but primary cell and organoids have the enormous advantage of been rapidly analyzable. Moreover, our results also highlight the fact that it is vital to perform a proper transcriptomic characterization to maintain the chemosensitivity traceability and correctly interpret results. In agreement with us, previous studies on gemcitabine [30], carfilzomib [24] and folfirinox [15] have shown similar findings, unraveling complex multigene signatures to predict chemosensitivity tuning the final output through a cross-model validation approach. This work suggests that inter-model transcriptomic harmonization following the chemosensitivity profile is a key factor in determining the clinical translatability of results obtained with these models. Chemosensitivity phenotype dependence was confirmed through pathways analysis, where the model polarization underlay the molecular subtype, determining the sensitive/resistant enrichment. Despite the method limitations related to pathway redundancy and gene variability bias, we observed a high concordance between the results obtained with the different models. XDPO displayed an increased enrichment to sensitive pathways, whereas XDPCC showed a resistant profile. Therefore, PDX was the most representative model with a wider phenotype range

## 5. Conclusions

In conclusion, we describe for the first time a detailed comparison between 3 different types of patient-derived PDAC preclinical models at the transcriptomic and chemosensitivity level. We established the basal-like/classical frame as a strong factor determining drug response independently of the model. Furthermore, we highlighted the relevance of validating and refining predictive transcriptomic signatures using a combination of these models. This fact is fundamental to achieve clinical applicability of the results obtained suggesting that these models are highly complementary and need to be used in a combinatory approach to achieve the best results.

## Data Availability

All datasets generated for this study are available upon reasonable request from the authors.

## Supplementary Materials

The following are available online at https://www.mdpi.com/article/10.3390/cancers13102473/s1, Figure S1: Unsupervised analysis with the 10,000 genes across all 3 models, Figure S2: Drug sensitivity in PDX models, Figure S3: Chemosensitivity profile of XDPO. Chemograms for XDPO treated with gemcitabine, irinotecan, 5-FU and oxaliplatin. Figure S4: Chemosensitivity profile of XDPCC. Chemograms of gemcita-bine, irinotecan, 5-FU and oxaliplatin. Figure S5: Boxplots comparing the CSS for all drugs tested for PDX, XDPO and XDPCC, Figure S6: Irinotecan chemosensitivity profile. a Correlation graph between the best component obtained from the ICA analysis and irinotecan CSS, Figure S7: 5-FU chemosensitivity profile. a Correlation graph between the best component obtained from the ICA analysis and 5-FU CSS, Figure S8: Oxaliplatin chemosensitivity profile, Figure S9: Global molecular pathways analysis, Table S1: Patient-derived xenografts (PDX), and xenograft-derived pancreatic organoids (XDPO), Table S2: Clinicopathological characteistics of patient cohort.
