# Supplementary material for: Exploring the Complementarity of Pancreatic Ductal Adenocarcinoma Preclinical Models
Source: Cancers (Basel). 2021 May 19;13(10):2473. doi: 10.3390/cancers13102473 (PMC8161239; doi:10.3390/cancers13102473)
Supplement: Supplementary file 1 [file cancers-13-02473-s001.zip › cancers-1201692-supplementary.pdf]

cancers

MDPI

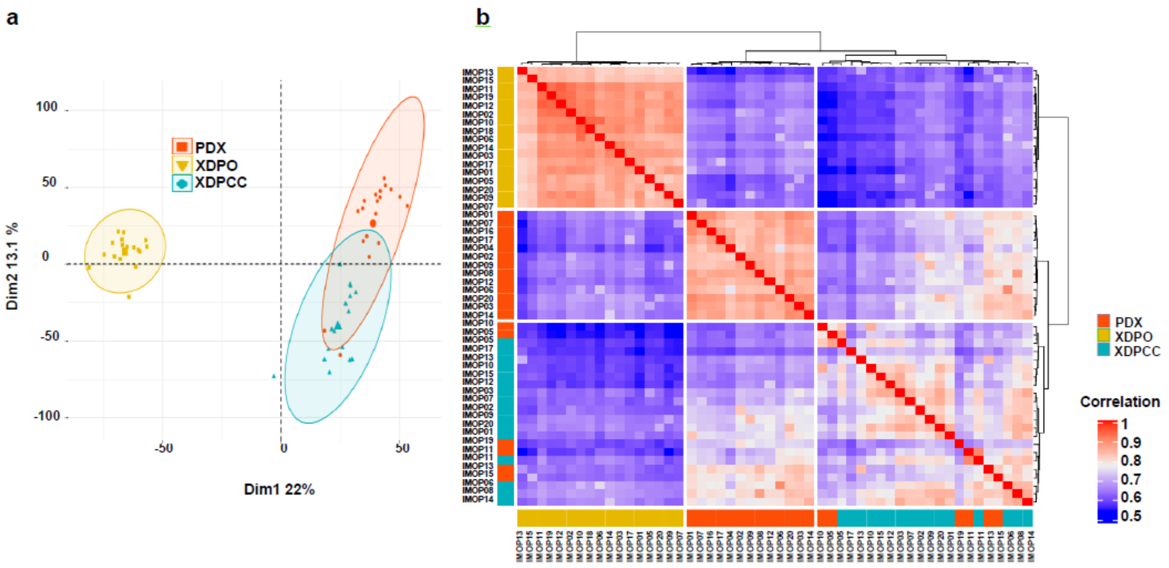

Figure S1: Unsupervised analysis with the 10,000 genes across all 3 models. a PCA plot performed with the top 10,000 more variable genes. b ComplexHeatmap with correlation matrix for all 3 models.

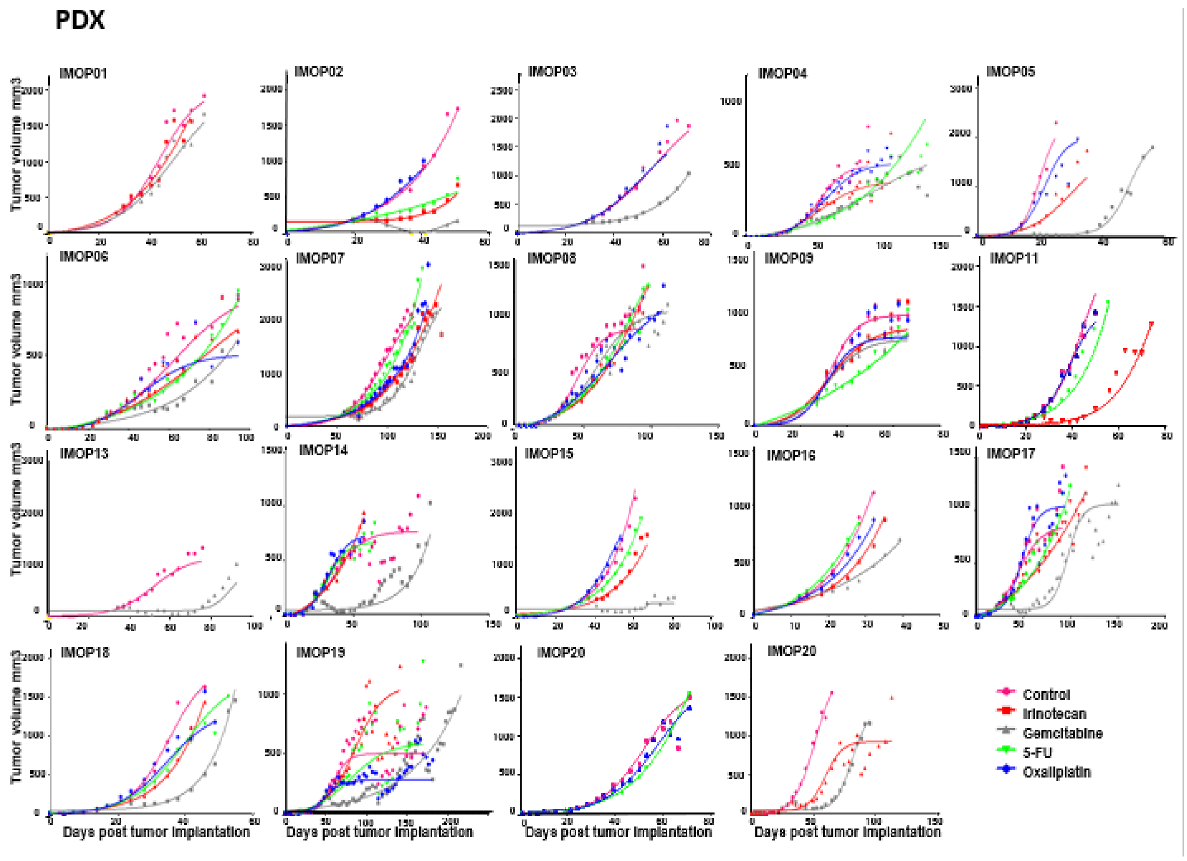

Figure S2: Drug sensitivity in PDX models. Growth curves of all PDX samples transplanted in the mice. The x-axis illustrates the time in days and y-axis increase in tumor volume (mm3).

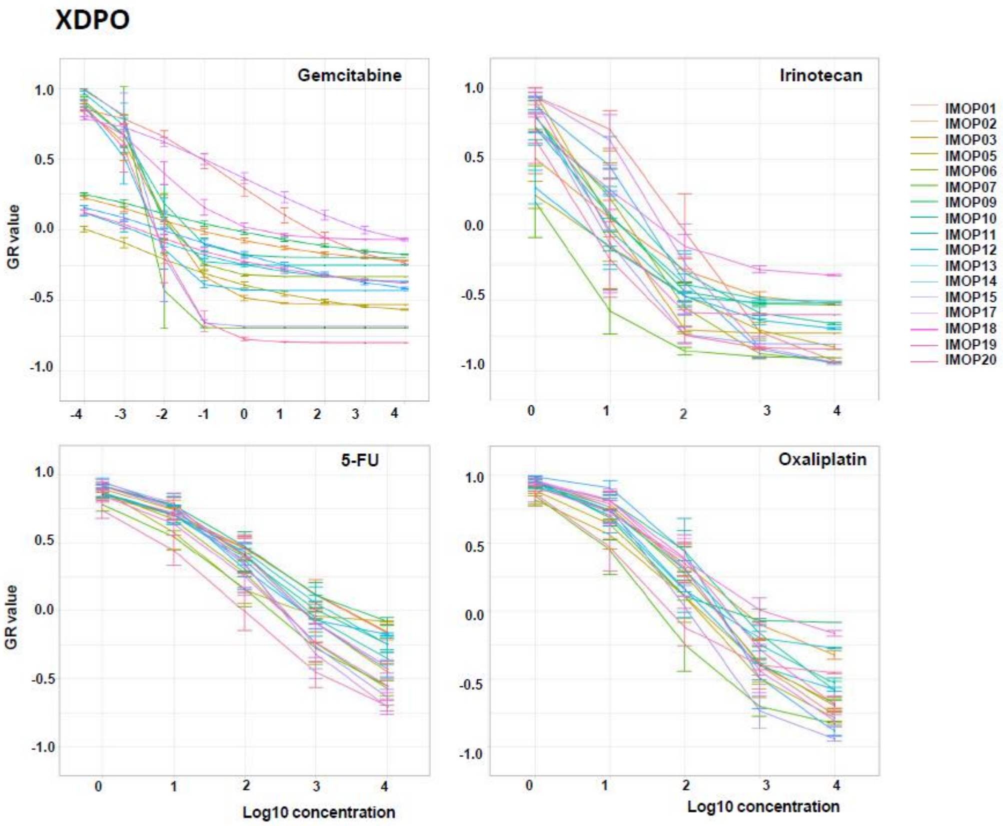

Figure S3: Chemosensitivity profile of XDPO. Chemograms for XDPO treated with gemcitabine, irinotecan by its metabolite SN38, 5-FU, and oxaliplatin. Error bars are calculated based on the differences in chemosensitivity in-between all of the biological replicates. The y-axis illustrates the Grow Rate (GR) values and the x-axis increasing concentrations of each drug on a Log10 scale.

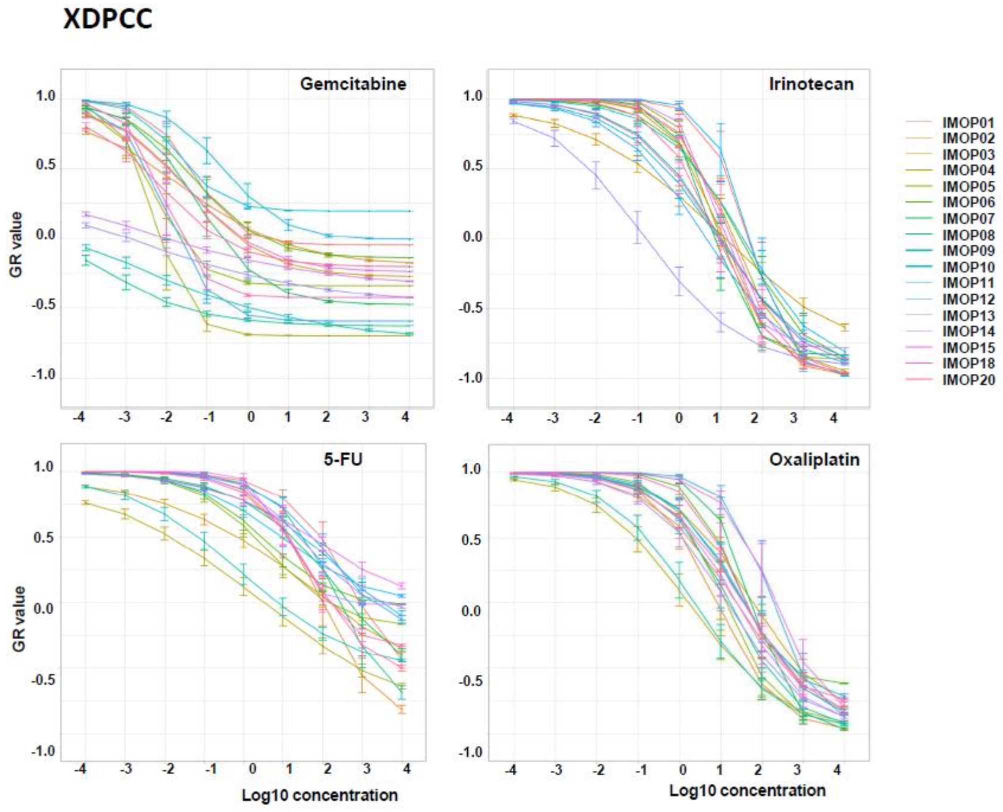

Figure S4: Chemosensitivity profile of XDPCC. Chemograms of gemcitabine, irinotecan by its metabolite SN38, 5-FU, and oxaliplatin. Error bars are calculated based on the differences in chemosensitivity in-between all of the biological replicates. The y-axis illustrates the Grow Rate (GR) values and x-axis increasing concentrations of each drug on a Log10 scale.

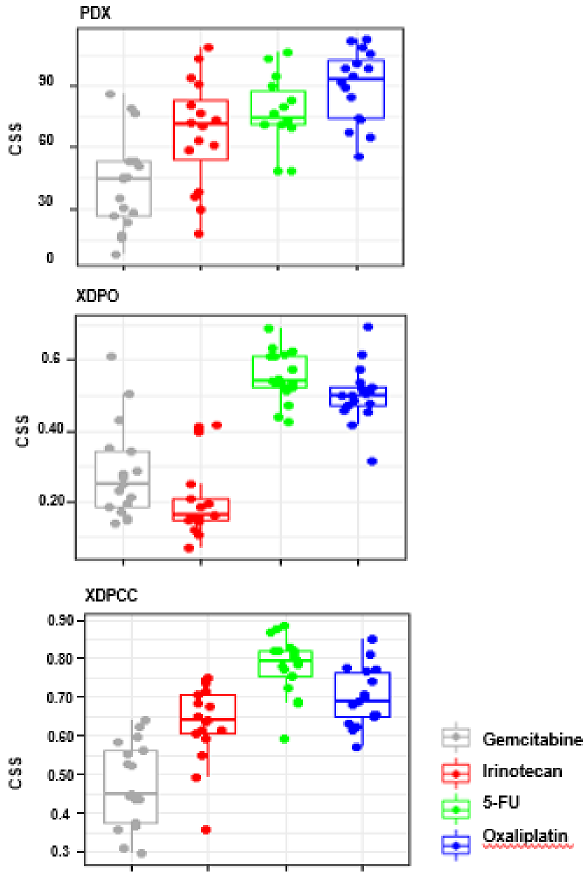

Figure S5: Boxplots comparing the CSS for all drugs tested for PDX, XDPO and XDPCC.

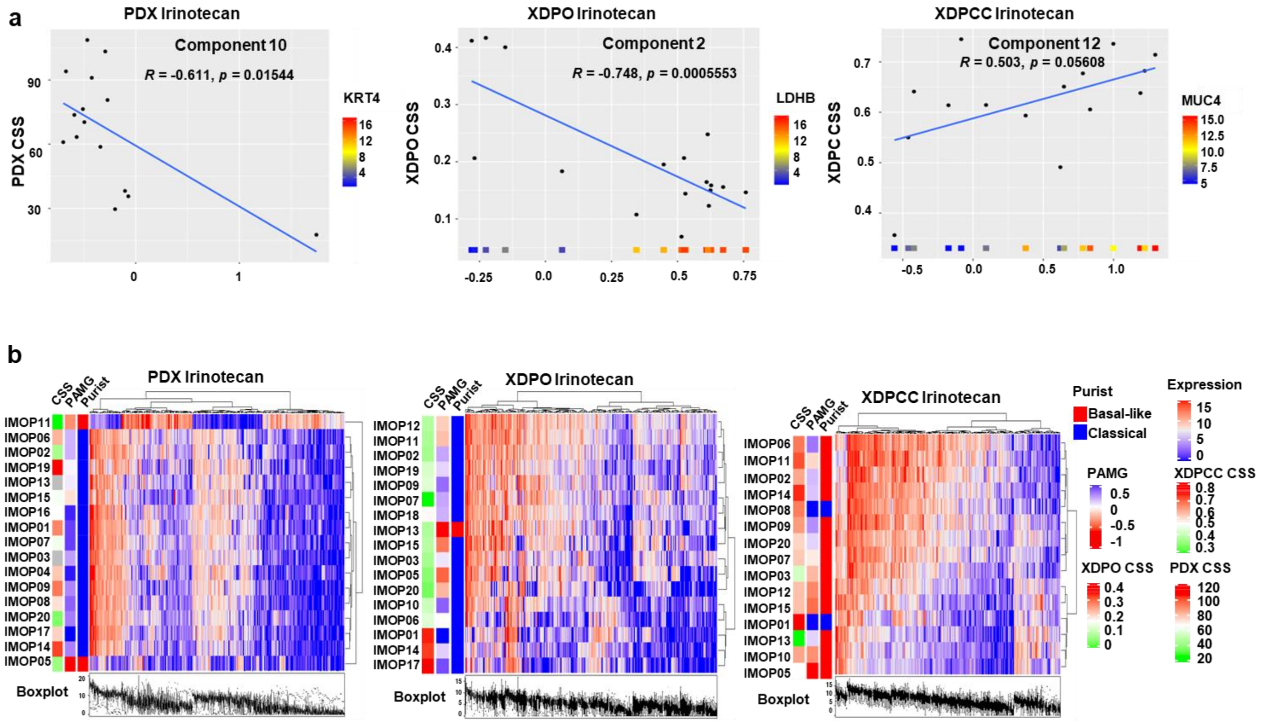

Figure S6: Irinotecan chemosensitivity profile. a Correlation graph between the best component obtained from the ICA analysis and irinotecan CSS. The CSS is displayed on the y-axis and the contribution of the witness gene of the x-axis. b Complexheatmaps illustrating the association of each profile with the chemosensitivity and phenotype.

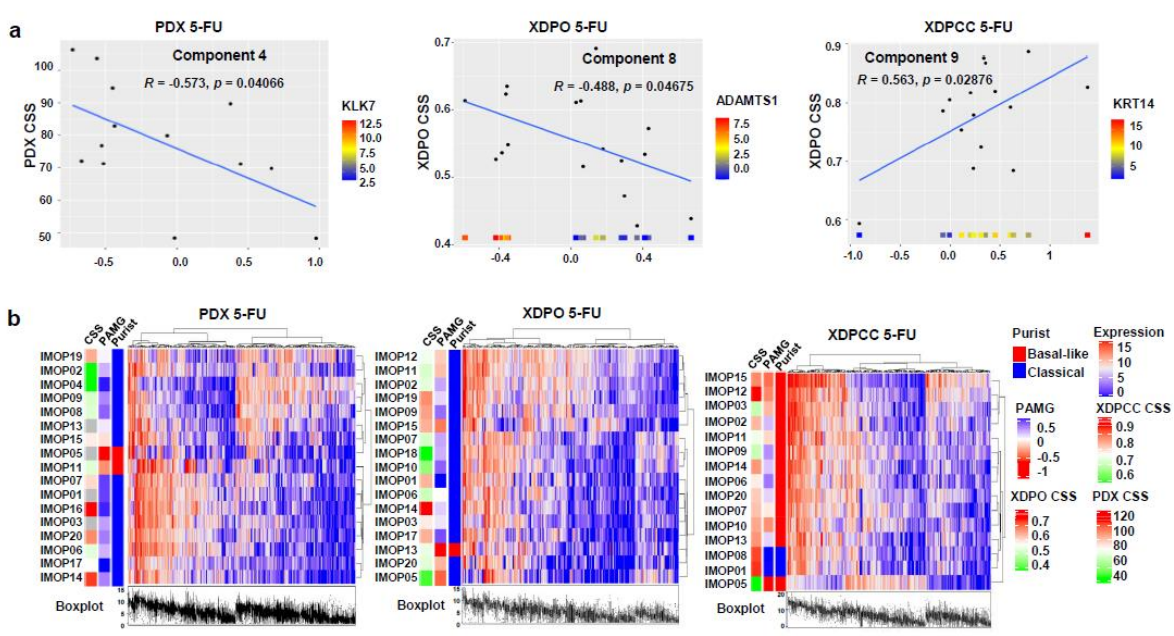

Figure S7: 5-FU chemosensitivity profile. a Correlation graph between the best component obtained from the ICA analysis and 5-FU CSS. The CSS is displayed on the y-axis and the contribution of the witness gene of the x-axis. b Complex heat maps illustrating the association of each signature with sensitivity and phenotype.

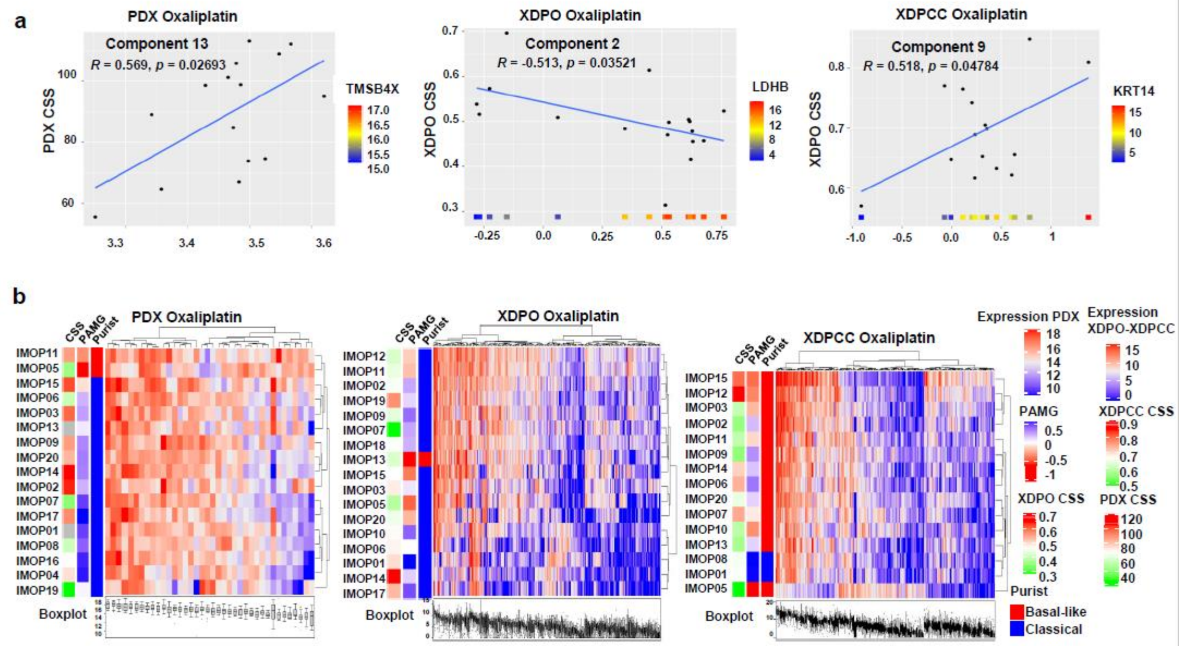

Figure S8: Oxaliplatin chemosensitivity profile. a Correlation graph between the best component obtained from the ICA analysis and Oxaliplatin CSS. The CSS is displayed on the y-axis and the contribution of the witness gene of the x-axis. b Complexheatmaps illustrating the association of each profile with sensitivity and phenotype.

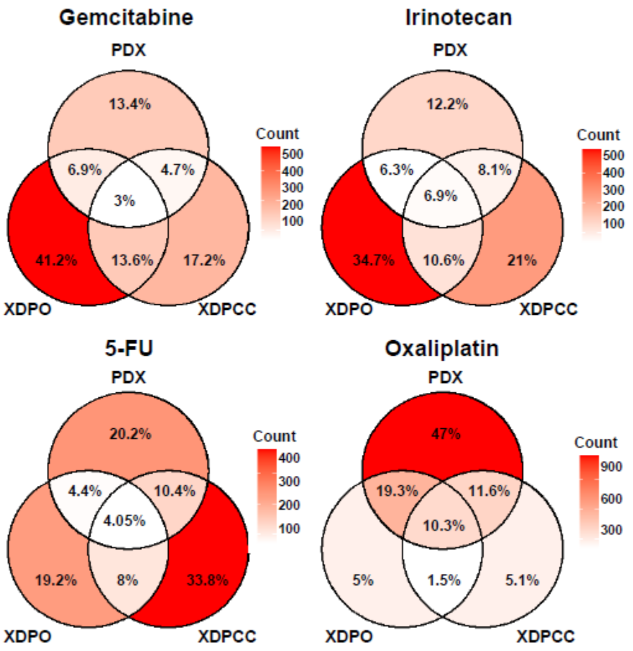

Figure S9: Global molecular pathways analysis. Venn diagram of all of the molecular pathways in common with all three models were generated. Count legend to the right depicts the number of pathways in common between models. The percentages are also included.

Table S1: Patient-derived xenografts (PDX),and xenograft-derived pancreatic organoids (XDPO)

PDX					
ID	Gemcitabine	Irinotecan	5-FU	Oxaliplatin	RNAseq
IMOP01	IMOP01	IMOP01	NA	NA	IMOP01
IMOP02	IMOP02	IMOP02	IMOP02	IMOP02	IMOP02
IMOP03	IMOP03	NA	NA	IMOP03	IMOP03
IMOP04	IMOP04	IMOP04	IMOP04	IMOP04	IMOP04
IMOP05	IMOP05	IMOP05	NA	IMOP05	IMOP05
IMOP06	IMOP06	IMOP06	IMOP06	IMOP06	IMOP06
IMOP07	IMOP07	IMOP07	IMOP07	IMOP07	IMOP07
IMOP08	IMOP08	IMOP08	IMOP09	IMOP08	IMOP08
IMOP09	IMOP09	IMOP09	IMOP09	IMOP09	IMOP09
IMOP10	NA	NA	NA	NA	IMOP10
IMOP11	NA	IMOP11	IMOP11	IMOP11	IMOP11
IMOP12	NA	NA	NA	NA	IMOP12
IMOP13	IMOP13	NA	NA	NA	IMOP13
IMOP14	IMOP14	IMOP14	IMOP14	IMOP14	IMOP14
IMOP15	IMOP15	IMOP15	IMOP15	IMOP15	IMOP15

IMOP16	IMOP16	IMOP16	IMOP16	IMOP16	IMOP16
IMOP17	IMOP17	IMOP17	IMOP17	IMOP17	IMOP17
IMOP18	IMOP18	IMOP18	IMOP18	IMOP18	NA
IMOP19	IMOP19	IMOP19	IMOP19	IMOP19	IMOP19
IMOP20	IMOP20	IMOP20	IMOP20	IMOP20	IMOP20
N=20	N=17	N=16	N=14	N=16	N=19

XDPO					
ID	Gemcitabine	Irinotecan	5-FU	Oxaliplatin	RNAseq
IMOP01	IMOP01	IMOP01	IMOP01	IMOP01	IMOP01
IMOP02	IMOP02	IMOP02	IMOP02	IMOP02	IMOP02
IMOP03	IMOP03	IMOP03	IMOP03	IMOP03	IMOP03
IMOP04	NA	NA	NA	NA	NA
IMOP05	IMOP05	IMOP05	IMOP05	IMOP05	IMOP05
IMOP06	IMOP06	IMOP06	IMOP06	IMOP06	IMOP06
IMOP07	IMOP07	IMOP07	IMOP07	IMOP07	IMOP07
IMOP08	NA	NA	NA	NA	NA
IMOP09	IMOP09	IMOP09	IMOP09	IMOP09	IMOP09
IMOP10	IMOP10	IMOP10	IMOP10	IMOP10	IMOP10
IMOP11	IMOP11	IMOP11	IMOP11	IMOP11	IMOP11
IMOP12	IMOP12	IMOP12	IMOP12	IMOP12	IMOP12
IMOP13	IMOP13	IMOP13	IMOP13	IMOP13	IMOP13
IMOP14	IMOP14	IMOP14	IMOP14	IMOP14	IMOP14
IMOP15	IMOP15	IMOP15	IMOP15	IMOP15	IMOP15
IMOP16	NA	NA	NA	NA	NA
IMOP17	IMOP17	IMOP17	IMOP17	IMOP17	IMOP17
IMOP18	IMOP18	IMOP18	IMOP18	IMOP18	IMOP18
IMOP19	IMOP19	IMOP19	IMOP19	IMOP19	IMOP19
IMOP20	IMOP20	IMOP20	IMOP20	IMOP20	IMOP20
N=20	N=17	N=17	N=17	N=17	N=17

Table S2: Clinicopathological characteistics of patient cohort.

Patient ID	Sexe	Resectab	Age at diag.	Tumor loc.	Resection margin	Differentiation	Tumor status at diag.	Specimen type
IMOP01	Male	Resect.	61	Body	R0	Well diff.	Loc.	Primary tum.
IMOP02	Male	Resect.	87	Tail	R0	Undiff.	Loc.	Primary tum.
IMOP03	Male	Resect.	73	Tail	R0	Unk.	Unk	Primary tum.
IMOP04	Female	Resect.	50	Body	R0	Moderately diff.	Loc.	Primary tum.
IMOP05	Male	Resect.	57	Head	R0	Undiff	Loc.	Primary tum.
IMOP06	Male	Resect.	68	Head	R0	Well diff.	Loc.	Primary tum.
IMOP07	Male	Resect.	57	Head	R1	Undiff.	Loc.	Primary tum.
IMOP08	Male	Unresect.	41	Tail	NA	Unk.	Metast.	Primary tum.
IMOP09	Male	Resect.	72	Tail	R0	Well diff.	Loc.	Primary tum.
IMOP10	Male	Unresect.	66	Head	NA	Unk.	Metast.	Primary tum.
IMOP11	Female	Unresect.	52	Unk	NA	Unk.	Metast.	Liver meta.
IMOP12	Male	Unresect.	68	Head	NA	Unk.	Loc. Adv	Primary tum.
IMOP13	Female	Unresect.	66	Head	NA	Unk.	Metast.	Primary tum.
IMOP14	Male	Resect.	74	Head	R0	Moderately diff.	Loc.	Primary tum.
IMOP15	Female	Resect.	62	Head	R0	Moderately diff.	Loc.	Primary tum.
IMOP16	Female	Resect.	80	Tail	R1	Moderately diff.	Loc.	Primary tum.
IMOP17	Male	Resect.	78	Head	R0	Well diff.	Loc.	Primary tum.
IMOP18	Female	Resect.	73	Head	R0	Moderately diff.	Loc.	Primary tum.
IMOP19	Female	Resect.	68	Tail	R0	Moderately diff.	Loc.	Primary tum.
IMOP20	Male	Unresect.	77	Unk	NA	Well diff.	Metast.	Liver meta
